# Effect of pharmacist-led intervention protocol on preventing postoperative delirium after elective cardiovascular surgery

**DOI:** 10.1371/journal.pone.0292786

**Published:** 2023-10-12

**Authors:** Yuki Asai, Tatsuki Yanagawa, Masaaki Takahashi

**Affiliations:** Pharmacy, National Hospital Organization Mie Chuo Medical Center, Hisaimyojin, Tsu, Mie, Japan; Radiation Effects Research Foundation, JAPAN

## Abstract

Postoperative delirium (PD) is an acute brain dysfunction, with a particularly high incidence after cardiovascular surgery. Pharmacist-led interventions show limited evidence in attenuating PD in cardiovascular surgery. In this retrospective cohort study, we aimed to clarify the risk factors of PD for cardiovascular surgery focused on pharmacotherapy and elucidate the effect of pharmacist-led intervention on the PD attenuation rate based on protocol-based pharmaceutical management (PBPM). This study included 142 adult patients who underwent elective valve replacement or valvuloplasty. The risk factors for PD were investigated using multivariate logistic regression analysis. Taking risk factors into consideration, a protocol was developed to discontinue benzodiazepines prescriptions by ward pharmacists, and replace with ramelteon and suvorexant if all the following factors apply: 1) number of medications ≥ 6 drugs, 2) number of doses to take ≥ 4 times, and 3) regular use of benzodiazepines or insomnia. Subsequently, the PD rate was compared during a period of two years and 6 months between the pre-PBPM (n = 39) and post-PBPM (n = 62). The PD rate for elective valve replacement or valvuloplasty was 25% (35/142). The adjusted odds ratio for polypharmacy was 3.3 (95% confidence interval: 1.2–8.9, *p* = 0.016), suggesting that preoperative risk assessment may be essential for patients with polypharmacy. The PD rate significantly decreased to 13% (8/62) in the post-PBPM group compared with 33% (13/39) in the pre-PBPM group (*p* = 0.014). There was a significant decrease in benzodiazepines use in post-PBPM compared with pre-PBPM (*p* = 0.026); however, the rate of ramelteon and orexin receptor antagonists use increased by PBPM introduction (*p* < 0.001). Although the present PBPM still requires further modification, it is simple and potentially useful for pharmacists to assess the risk of patients undergoing any elective cardiovascular surgery.

## 1 Introduction

Postoperative delirium (PD) is a complication associated with long hospitalization, excessive medical costs, and poor prognosis, and is defined as acute brain dysfunction [1]. Cardiovascular surgery has been reported to be associated with a high incidence of PD, ranging from 5 to 39% [2], and PD caused by cardiovascular surgery affects the 30-day and long-term mortality [3]. PD commonly occurs within 3 days after surgery [4], therefore, it is crucial for healthcare workers to assess the risk and conduct interventions to prevent PD before surgery.

It has been well-known that risk factors for delirium were categorized as predisposing, promoting, or direct factors [5]. Pain and bedside environmental control were promoting factors; however, medication has been recognized as direct factor, suggesting that medication may be relatively more important than those of promoting factors. Recently, a network meta-analysis revealed that medications, including benzodiazepines, may be strongly associated with PD, and pharmacological interventions may be involved in PD attenuation [6]. Therefore, the development of PD may be controlled by pharmacists, who propose reduction or discontinuation of drugs associated with the risk of PD.

In the United States, pharmacists in collaboration with physicians would legally prescribe medications, perform blood tests, and change dosages [7]. Protocol-based pharmaceutical management (PBPM), where pharmacists participate in pharmacological therapy based on an agreement protocol with the physician, has been established as an alternative system in Japan. PBPM has spread throughout Japan with demonstrated clinical efficacy [8–10]; therefore, it is possible to improve PD in cardiovascular surgery by preoperative assessment of the status of medication for high-risk patients and intervention by a pharmacist using PBPM. However, there is little evidence regarding pharmacist-led protocol development and evaluation of the efficacy of PD risk factors in cardiovascular surgery.

Herein, two surveys were conducted to evaluate the effectiveness of protocol-based pharmacist interventions in the prevention of PD in cardiovascular surgery. The initial stage aimed to identify predictors of PD that could be readily identified by pharmacists. In the second stage, examination of the effects of reducing benzodiazepines, and incorporating ramelteon and orexin receptor antagonists on PD rate of high-risk patients was identified based on the predictors.

## 2 Martials and methods

### 2.1 Study design

**Fig 1:**
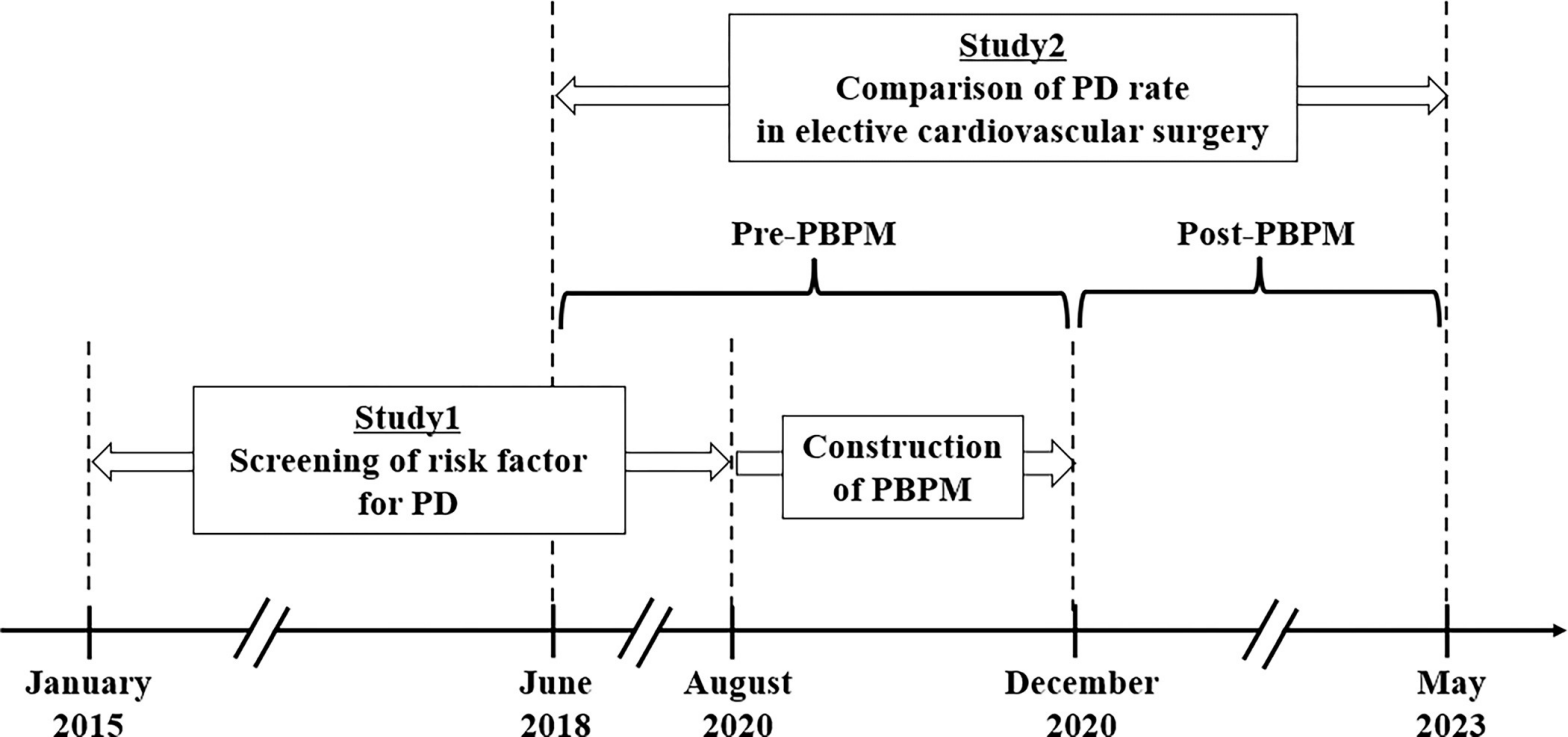
Schematic illustrating the study procedures.

The study design scheme is illustrated in Fig 1. A single-center retrospective cohort study (study 1 and study 2) was conducted at the National Hospital Organization Mie Chuo Medical Center (Mie, Japan) using electronic medical records. In study 1, to clarify the risk factors for PD in cardiovascular surgery focused on pharmacotherapy, a retrospective cohort study was conducted using clinical data from patients who had undergone valve replacement or valvuloplasty from January 1, 2015, to August 31, 2020. Based on the identified risk factors of PD, a protocol was developed in consultation with a cardiovascular surgeon. In study 2, two years and six months after PBPM implementation, its efficacy was evaluated in a retrospective cohort study between pre-PBPM (June 1, 2018, to November 30, 2020) and post-PBPM (December 1, 2020, to May 31, 2023).

### 2.2 Screening the PD risk factors: Study 1

#### 2.2.1 Subjects

We focused on valve replacement or valvuloplasty because of the high incidence of PD [11], and the frequency of these operations at Mie Chuo Medical Center. The primary outcome was the onset of delirium up to 7 days after surgery. Based on the primary outcome, the inclusion criterion was hospitalization of adult patients (age ≥18 years) for elective valve replacement or valvuloplasty. Therefore, the following patients were excluded from the analysis: (a) patients aged <18 years, (b) those who underwent emergency surgery, and (c) those who died during or within seven days after surgery. When a patient had a history of multiple surgeries, the first episode was used for analysis.

#### 2.2.2 Data collection and analysis

Delirium was defined using the Intensive Care Delirium Screening Checklist (ICDSC) as an ICDSC score ≥ 4 (sensitivity, 99%; specificity, 64%) [12]. Scoring was performed according to physician or nurse records, as in previous studies [13–15]. ICDSC scoring was performed once a day, starting on the first postoperative day. We classified the patients into delirium and non-delirium groups.

Information regarding the patients’ basic characteristics, comorbidity, and preoperative oral medications was collected before surgery. For comorbidity, we investigated a history of reported risk factors for PD in cardiovascular surgery, such as anemia [16], atrial fibrillation [16], cerebral infraction [17], diabetes mellitus [18], heart failure [19], and major depression [16]. Additionally, the number of comorbidities was calculated by totaling the number of underlying diseases listed above for each patient. Information on preoperative oral medications was obtained from the hospital upon admission. Polypharmacy was defined as the use of six or more drugs [20], the non-polypharmacy group included patients who took fewer than six drugs. The number of doses to take was referenced to the maximum timing of oral dosing, example, 3 after every meal, 4 after every meal, and before bed. The duration of intensive care unit (ICU) stay and ICDSC scores were evaluated for up to 7 days after surgery. Additionally, age, rate of benzodiazepine treatment, and duration of ICU stay were compared between the polypharmacy and non-polypharmacy groups.

### 2.3 Evaluation of PBPM efficacy: Study 2

#### 2.3.1 Protocol

**Fig 2:**
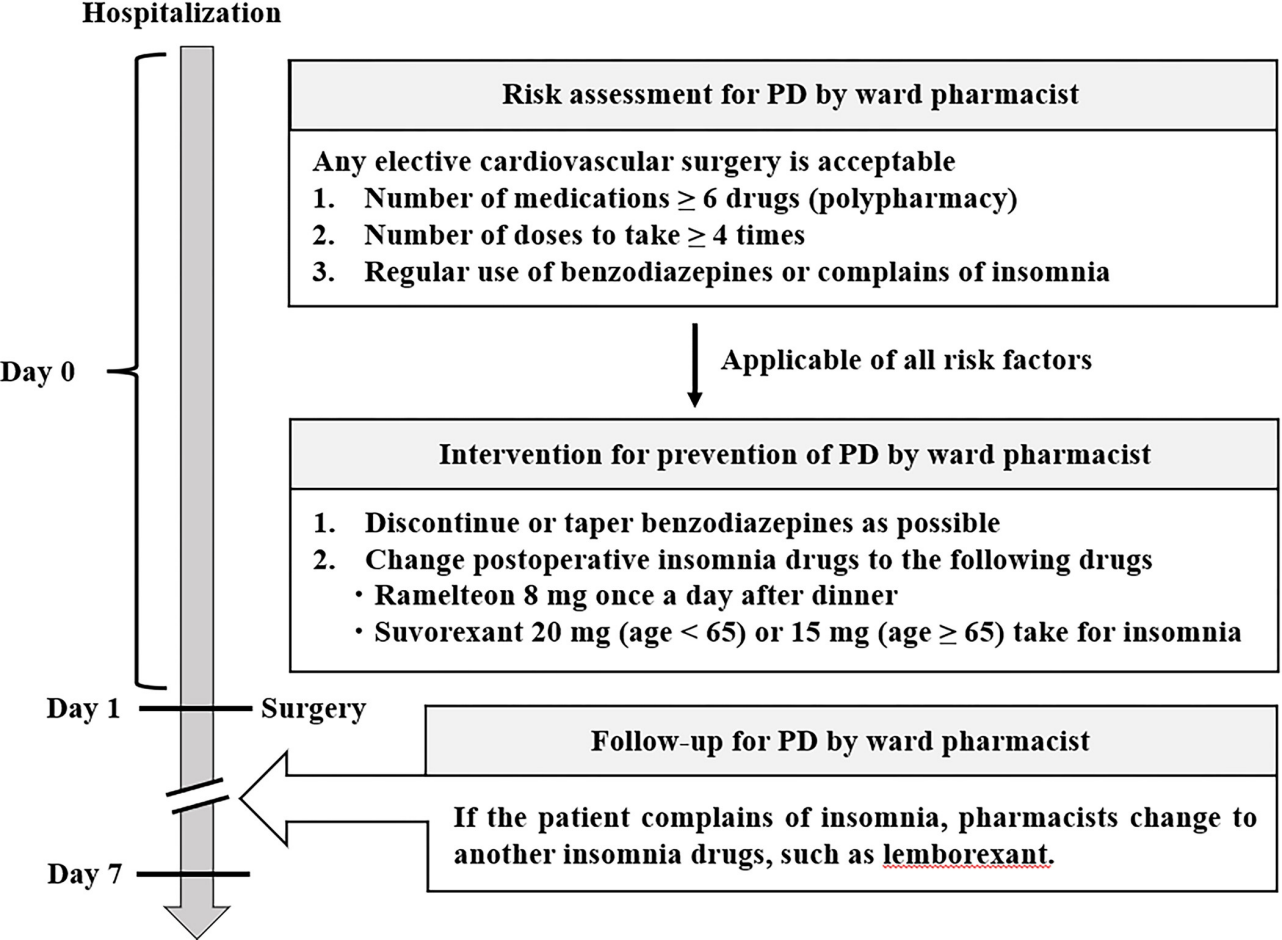
PBPM flow chart showing method of pharmacist-led intervention for preventing PD in elective cardiovascular surgery.

Pharmacists in charge of the cardiovascular ward and surgeons participated in this protocol at a secondary hospital in Japan, which was implemented from December 2020. The incidence of PD by procedure varied widely among reports [2], therefore, this protocol was applied to patients admitted to the cardiology unit for any elective cardiovascular surgery (Fig 2). On the day before surgery, pharmacists conducted a risk assessment from interviews with patients and medications brought to the hospital on admission: 1) number of medications ≥ 6 drugs, 2) number of doses to take ≥ 4 times, and 3) regular use of benzodiazepines or insomnia. The combination use of ramelteon and suvorexant may be involved in PD reduction [21], therefore, the pharmacist would have provided the following two interventions for patients who meet the three risk factors: 1) discontinue or taper benzodiazepines as soon as possible, and 2) change postoperative sleeping drugs (ramelteon 8 mg once a day after dinner [22] and suvorexant 20 mg (age < 65) or 15 mg (age ≥ 65) for insomnia). After surgery, the pharmacist assessed the patient’s sleep status, and changed to another sleeping drugs including lemborexant for up to 7 days after the surgery.

#### 2.3.2 Subjects

The inclusion criterion was adult patients (age ≥18 years) who fit the PBPM, therefore, the following patients were excluded from analysis: (a) aged < 18 years, (b) underwent emergency surgery, (c) number of medications < 6 drugs, (d) number of doses to take < 4 times, (e) discharge within 7 days after surgery, and (f) death during surgery or death within 7 days after surgery.

#### 2.3.3 Data collection and analysis

The primary outcome was the onset of delirium up to 7 days after surgery in all eligible patients. Additionally, since PD risk factors in patients undergoing valve replacement or valvuloplasty was identified in study 1, we compared the incidence of PD in patients who underwent open-heart surgery (coronary artery bypass grafting, aortic root replacement and valve replacement/valvuloplasty) between pre- and post-PBPM as a subgroup analysis. The secondary outcome was the rate of benzodiazepine, ramelteon, orexin receptor antagonists (suvorexant or lemborexant) use up to 7 days after surgery.

### 2.4 Statistical analysis

In study 2, to compare the PD rate by PBPM implementation, a sample size of 34 patients was recruited to each group for analysis with a detection power of 0.8 and a significance level of 0.05, according to a previous study showing that intervention by healthcare workers reduced the PD rate from 38% to 9.4% in high-risk patients for PD [23]. Univariate analyses were performed for the continuous and categorical variables. Differences in continuous variables were analyzed with Student’s *t-*test if they followed a normal distribution, and with the Mann–Whitney *U* test if they followed an abnormal distribution. Categorical variables were compared using the chi-square (χ^2^) test. Fisher’s exact test included one cell with an expected value of < 5 in a 2×2 contingency table. The objective variable was PD in the multivariate logistic regression analysis. As age [24], benzodiazepines [6], and ICU stay [24] may influence the onset of PD in cardiovascular surgery, these factors were selected as explanatory variables. The Hosmer–Lemeshow test was used to assess the goodness of fit of the multivariate logistic regression model, which was set at *p* > 0.05. Correlations between the number of medications and the number of doses to take were analyzed using Spearman’s correlation coefficient. Additionally, the correlation between the number of medications and the number of comorbidities were investigated. Multicollinearity was also evaluated using the variance inflation factor. The cutoff value was evaluated based on the sensitivity, specificity, and area under the curve (AUC) using receiver operating characteristic (ROC) analysis. All statistical analyses were performed using SPSS Statistics version 27 (IBM Japan, Tokyo, Japan), and the significance level was set at *p* < 0.05.

### 2.5 Ethics approval statement

This study was conducted in accordance with the Ethical Guidelines for Medical and Health Research Involving Human Subjects. The study protocol was approved by the National Hospital Organization Mie Chuo Medical Center (approval ref. MCERB-202244). The intervention based on PBPM was explained to the patients and their consent was obtained. Due to the retrospective study design, oral agreement was not required. Therefore, informed consent was obtained using an opt-out document posted on the website of the National Hospital Organization Mie Chuo Medical Center. We collected the clinical data of eligible patients on June 1, 2023. We had access to information that could identify individual participants after data collection.

## 3 Results

### 3.1 Screening the risk factors of PD: Study 1

#### 3.1.1 Subjects

A total of 142 of the 147 patients who underwent valve replacement or valvuloplasty between January 2015 and August 2020, were included in the analysis, since 5 patients died during surgery or within 7 days after surgery.

#### 3.1.2 Outcomes

**Fig 3:**
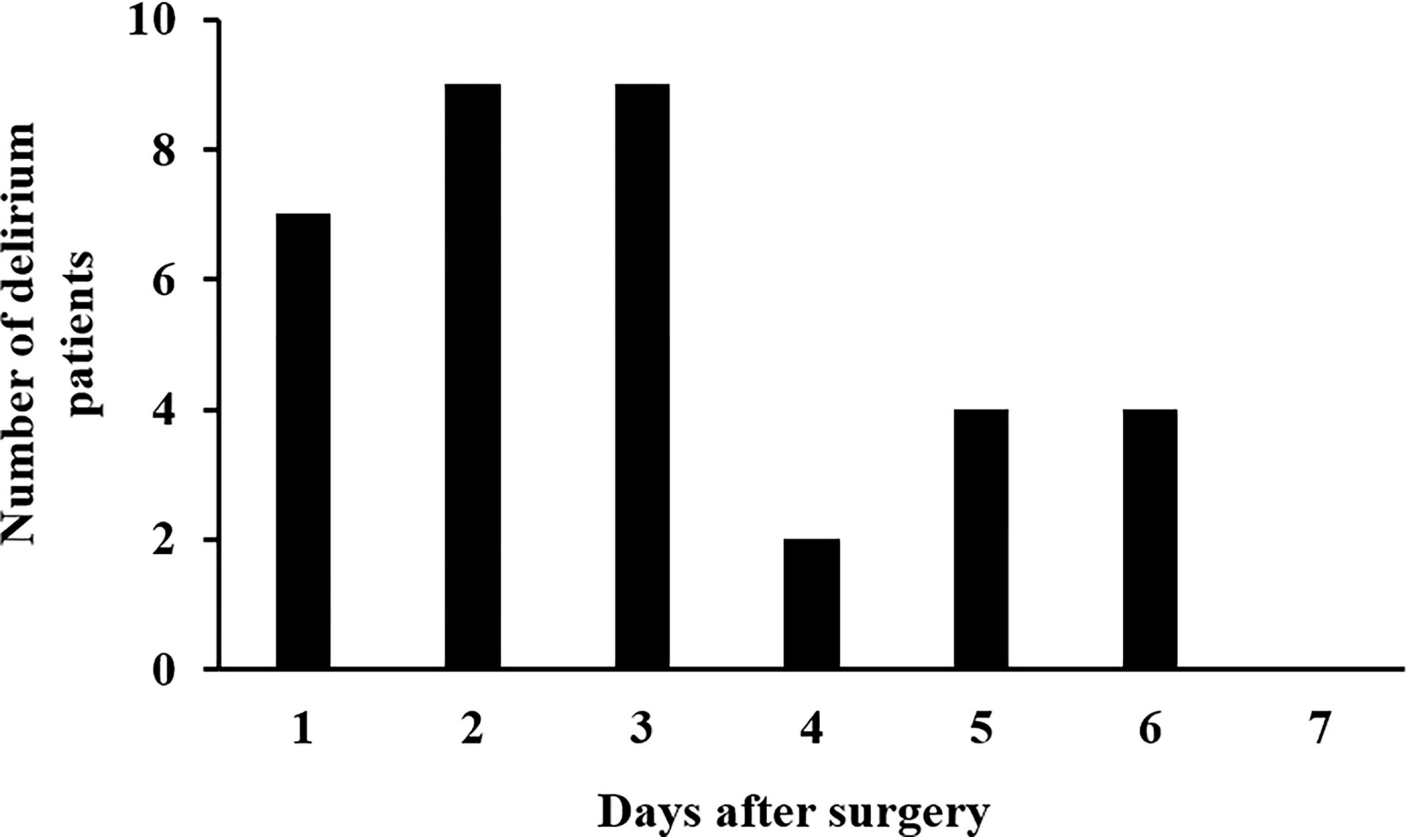
Time-dependent frequency of PD after valve replacement or valvuloplasty.

**Fig 4:**
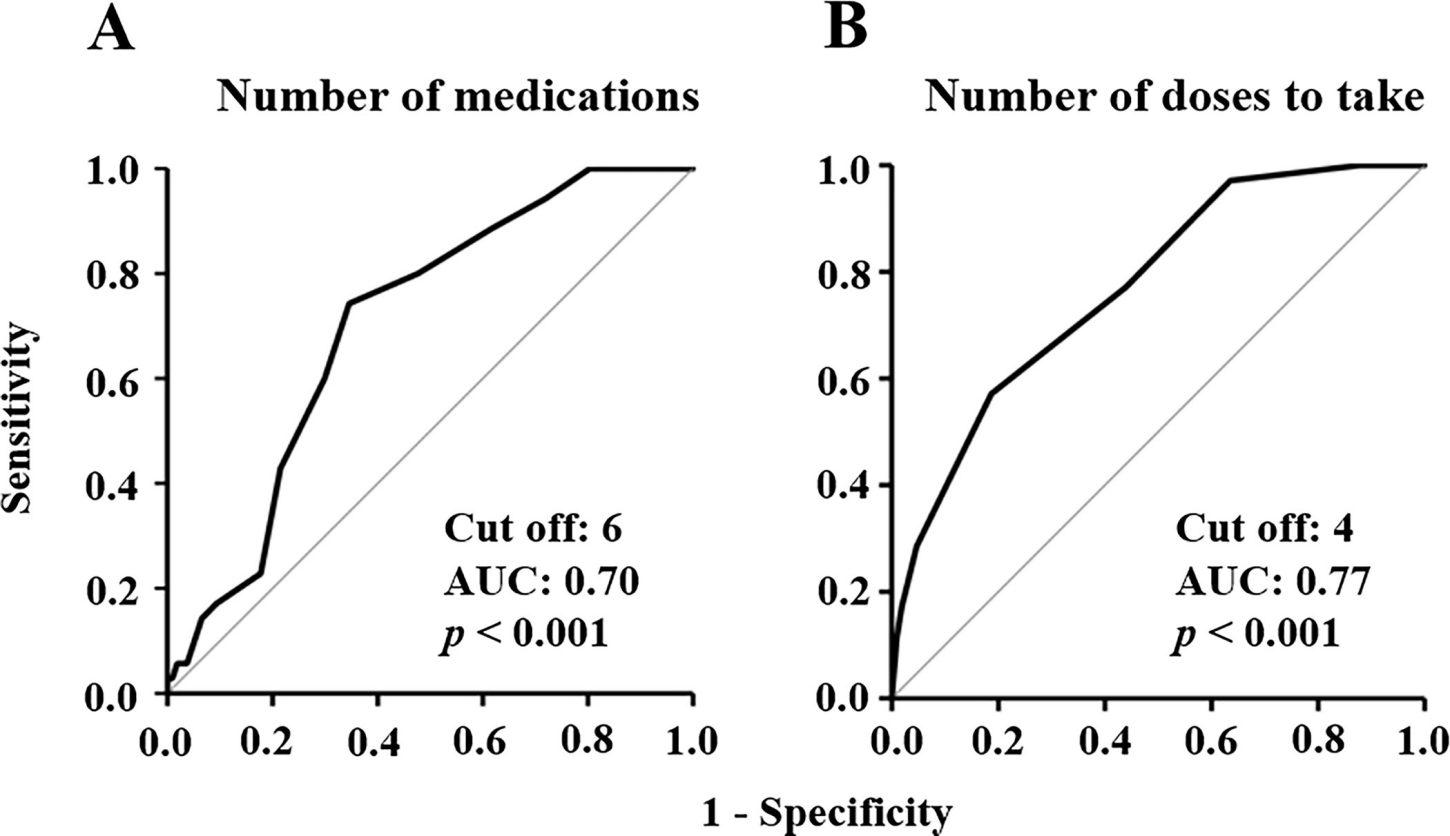
ROC curve of number of medications (A) and number of doses to take (B) for PD after valve replacement or valvuloplasty.

The incidence of PD was 25% (35/142 patients). The baseline characteristics of the patients are presented in Table 1. As shown in Fig 3, 71% (25/35) of PD occurred within 3 days after surgery. Univariate analyses revealed differences in age (*p* = 0.043), number of comorbidities (*p* = 0.014), cerebral infraction (*p* = 0.043), number of medications (*p* < 0.001), benzodiazepine treatment (*p* = 0.014), number of doses to take (*p* < 0.001), and duration of ICU stay (*p* = 0.004) between the delirium and non-delirium groups. There was a positive correlation between the number of medications and the number of doses to take (*r* = 0.70, *p* < 0.01), therefore, the number of doses to take was not selected as an explanatory variable for the multivariate logistic regression analysis. Multivariate logistic regression analysis revealed that the adjusted odds ratio for polypharmacy was 3.3 (95% confidence interval: 1.2–8.9, *p* = 0.016) (Table 2). The Hosmer–Lemeshow test calculated the *p* value for the goodness of fit of the multivariate logistic regression as 0.88. The values of variance inflation factor for age, number of comorbidities, number of medications, and duration of ICU stay were 1.2, 1.3, 1.5, and 1.1, respectively. Age, ICU stay, and benzodiazepine administration rates were significantly higher in the polypharmacy group than in the non-polypharmacy group (*p* < 0.001 for the three factors). The cut-off values for the number of medications and the number of doses to take were 6 (sensitivity, 0.74; specificity, 0.65; AUC, 0.70) and 4 (sensitivity, 0.57; specificity, 0.81; AUC, 0.77), respectively (Fig 4). A significant moderate correlation was observed between the number of medications and the number of comorbidities (*r* = 0.44, *p* < 0.001) (S1 Fig). The ROC analysis revealed that the cut-off value of the number of comorbidities was 2 (sensitivity, 0.43; specificity, 0.72; AUC, 0.63) (S2 Fig). A protocol was established based on these risk factors (Fig 2).

**Table 1:** Baseline clinical characteristics and laboratory data of eligible patients in valve replacement or valvuloplasty.

Factors	Non-delirium	Delirium	*p* value
	n = 107	n = 35	
Basic property			
Sex (Male/Female)	57/50	20/15	0.69^a^
Age (years)	72 (64, 77)^f^	76 (70, 79)^f^	0.043^d^
Body weight (kg)	56 (48, 66)^f^	56 (48, 60)^f^	0.22^d^
eGFR (mL/min)	66 ± 18^e^	65 ± 19^e^	0.85^c^
Albumin (g/dL)	2.8 (2.5, 3.0)^f^	2.7 (2.6, 2.9)^f^	0.44^d^
Total bilirubin (mg/dL)	0.9 (0.7, 1.2)^f^	0.9 (0.7, 1.3)^f^	0.69^d^
ALT (IU/L)	20 (15, 26)^f^	16 (13, 22)^f^	0.063^d^
Comorbidity			
Number of comorbidities	1 (1, 2)^f^	1 (1, 2)^f^	0.014^d^
Anemia, n (%)	4 (3.7)	2 (5.7)	0.64^b^
Atrial fibrillation, n (%)	15 (14)	10 (29)	0.050^a^
Cerebral infarction, n (%)	7 (6.5)	7 (20)	0.043^b^
Diabetes mellitus, n (%)	19 (18)	8 (23)	0.50^a^
Heart failure, n (%)	66 (62)	24 (69)	0.46^a^
Major depression, n (%)	2 (1.9)	2 (5.7)	0.25^b^
Preoperative oral medications			
Number of medications	4 (2, 7)^f^	7 (6, 8)^f^	< 0.001^d^
Poly pharmacy, n (%)	37 (35)	26 (74)	< 0.001^a^
Benzodiazepines, n (%)	12 (11)	10 (29)	0.014^a^
H_2_ blockers, n (%)	10 (9.3)	3 (8.6)	1.0^b^
Proton-pomp inhibitors, n (%)	22 (21)	8 (23)	0.77^a^
Steroids, n (%)	4 (3.7)	0 (0.0)	0.57^b^
*β* blockers, n (%)	26 (24)	13 (37)	0.14^a^
Anti-arrhythmic drugs, n (%)	6 (5.6)	4 (12)	0.25^b^
Anti-hypertensives, n (%)	63 (59)	21 (60)	0.91^a^
Anti-psychotics, n (%)	2 (1.9)	1 (2.9)	1.0^b^
Opioids, n (%)	1 (0.9)	0 (0.0)	1.0^b^
Number of doses to take	2 (1, 3)^f^	4 (3, 5)^f^	< 0.001^d^
Duration of ventilator management (day)	7 (4, 7)^f^	7 (6, 7)^f^	0.057^d^
Duration of ICU stays (day)	4 (3, 7)^f^	6 (4, 9)^f^	0.004^d^

ALT: Alanine aminotransferase. eGFR: Estimated glomerular filtration rate. ICU: Intensive care unit.

^a^Chi-square test

^b^Fisher’s exact test

^c^Student’s t-test

^d^Mann-Whitney U test

^e^Each value represents the mean±standard deviations

^f^Each value represents the median (25th, 75th percentile).

**Table 2:** Factors influencing the PD on multivariate logistic regression analysis.

Factors	Adjusted OR	95% CI	*p* value
Age	1.0	0.97–1.1	0.50
Benzodiazepine use	1.6	0.54–4.6	0.41
Number of comorbidities	1.3	0.77–2.3	0.31
Duration of ICU stay	1.1	0.96–1.2	0.20
Polypharmacy	3.3	1.2–8.9	0.016

OR: Odds ratio. ICU: Intensive care unit. 95% CI, 95% coefficient interval.

### 3.2 Evaluation of efficacy of PBPM: Study 2

#### 3.2.1 Subjects

**Fig 5:**
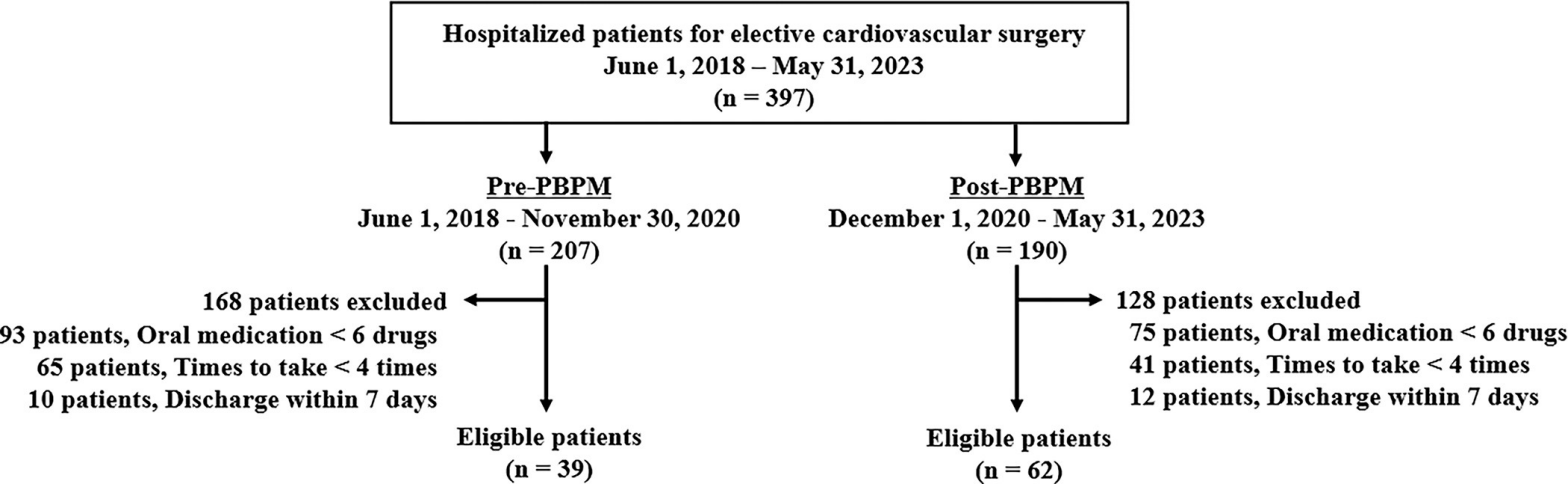
Flow diagram illustrating the patient selection process for study 2.

**Fig 6:**
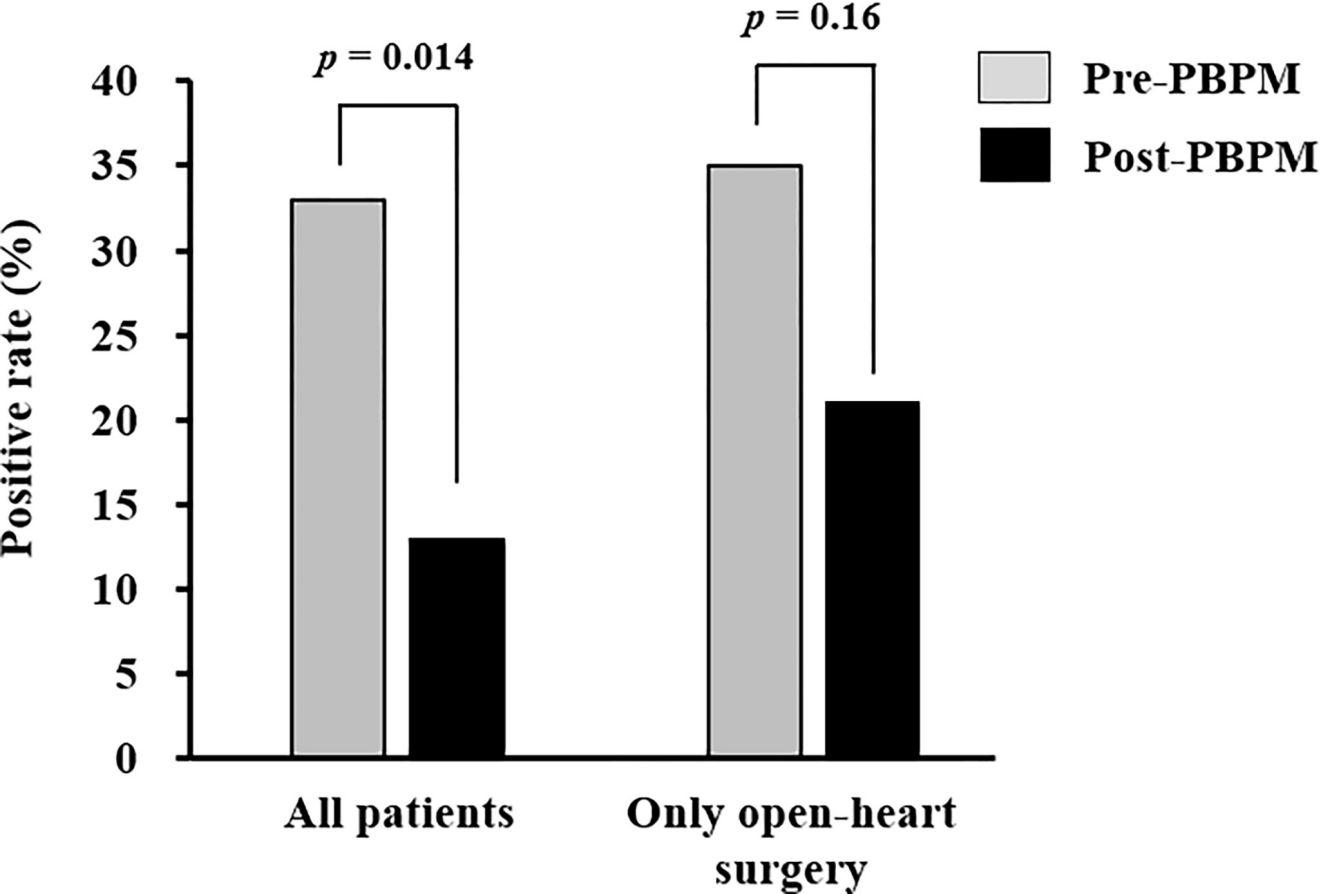
Effect of pharmacist-led intervention on the rates of PD.

We investigated 397 patients who were hospitalized for elective cardiovascular surgery between June 1, 2018, and May 31, 2023, and divided them into pre- (n = 207) and post-PBPM (n = 190) groups (Fig 5). After applying the inclusion and exclusion criteria, 39 and 62 patients were enrolled in the pre- and post-PBPM groups, respectively. The baseline clinical characteristics and laboratory data of patients during each period are shown in Table 3. Univariate analyses revealed differences in body weight (*p* = 0.009), total bilirubin level (*p* = 0.010), stent graft insertion rate (*p* = 0.002), and valve replacement or valvuloplasty rate (*p* < 0.001) between the pre- and post-PBPM groups. Additionally, patient backgrounds focusing on open-heart surgery are shown in S1 Table. As shown Fig 6, the PD rate was significantly decreased to 13% (8/62) by the PBPM compared with 33% (13/39) in the pre-PBPM group (*p* = 0.014) in all eligible patients. However, there was no significant difference in PD rate between pre- and post-PBPM (*p* = 0.158) in patients who underwent open-heart surgery. In the secondary outcome, a significant decrease in benzodiazepine use from 44% (17/39) to 23% (14/62) in post-PBPM compared with pre-PBPM, respectively (*p* = 0.026) (Table 3). The rate of ramelteon use significantly increased to 73% (45/62) in the post-PBPM compared with 31% (12/39) in the pre-PBPM (*p* < 0.001), and the rate of orexin receptor antagonists elevated from 13% (5/39) to 47% (29/62) (*p* < 0.001) (Table 3). Significant variations in the use of these insomnia drugs, except for benzodiazepines, were also observed when focusing on open-heart surgery patients (S1 Table).

**Table 3:** Baseline clinical characteristics and laboratory data of eligible patients in pre- and post-PBPM.

Factors	Pre-PBPM	Post-PBPM	*p* value
	n = 39	n = 62	
Preoperative Information			
Basic property			
Sex (Male/Female)	26/13	47/15	0.32^a^
Age (years)	76 (72, 80)^f^	75 (69, 80)^f^	0.42^d^
Body weight (kg)	57 (50, 60)^f^	61 (53, 69)^f^	0.009^d^
eGFR (mL/min)	62 ± 20^e^	59 ± 20^e^	0.44^c^
Albumin (g/dL)	3.0 (2.5, 3.7)^f^	2.8 (2.4, 3.4)^f^	0.30^d^
Total bilirubin (mg/dL)	0.9 (0.7, 1.3)^f^	0.7 (0.6, 0.9)^f^	0.010^d^
ALT (IU/L)	15 (11, 23)^f^	17 (11, 28)^f^	0.53^d^
Operation procedure			
CABG, n (%)	12 (31)	26 (42)	0.26^a^
Lower extremity bypass, n (%)	3 (7.7)	4 (6.5)	1.0^b^
Stent graft insertion, n (%)	2 (5.1)	19 (31)	0.002^a^
Aortic root replacement, n (%)	3 (7.7)	4 (6.5)	1.0^b^
Valve replacement/Valvuloplasty, n (%)	19 (49)	9 (15)	< 0.001^a^
Oral medications			
Number of medications	9 (7, 11)	9 (7, 11)	0.38^d^
Benzodiazepines, n (%)	16 (41)	18 (29)	0.21^a^
H_2_ blockers, n (%)	3 (7.7)	5 (8.1)	1.0^b^
Proton-pomp inhibitors, n (%)	13 (33)	37 (60)	0.010^a^
Steroids, n (%)	3 (7.7)	2 (3.2)	0.37^b^
*β* blockers, n (%)	12 (31)	29 (47)	0.11^a^
Anti-arrhythmic drugs, n (%)	7 (18)	3 (4.8)	0.043^b^
Anti-hypertensives, n (%)	31 (80)	54 (87)	0.31^a^
Dementia drugs, n (%)	0 (0.0)	1 (1.6)	1.0^b^
Anti-psychotics, n (%)	0 (0.0)	6 (9.7)	0.079^b^
Number of doses to take	4 (4, 5)	4 (4, 5)	0.60^d^
Postoperative Information			
Operation time (min)	290 (208, 405)^f^	275 (194, 347)^f^	0.24^d^
Anesthesia time (min)	367 (270, 467)^f^	332 (240, 422)^f^	0.16^d^
Duration of ventilator management (day)	6 (4, 7)^f^	5 (2, 7)^f^	0.17^d^
Duration of ICU stay (day)	4 (3, 4)^f^	3 (2, 5)^f^	0.20^d^
SOFA score	9 (5, 10)^f^	8 (3, 10)^f^	0.71^d^
Dexmedetomidine treatment, n (%)	21 (54)	30 (48)	0.59^a^
Insomnia drugs^g^ use			
Benzodiazepines, n (%)	17 (44)	14 (23)	0.026^a^
Ramelteon, n (%)	12 (31)	45 (73)	< 0.001^a^
Orexin receptor antagonists^h^, n (%)	5 (13)	29 (47)	< 0.001^a^

ALT: Alanine aminotransferase. CABG: Coronary artery bypass grafting. eGFR: Estimated glomerular filtration rate. ICU: Intensive care unit. SOFA: Sequential organ failure assessment.

^a^Chi-square test

^b^Fisher’s exact test

^c^Student’s t-test

^d^Mann-Whitney U test

^e^Each value represents the mean±standard deviations

^f^Each value represents the median (25th, 75th percentile)

^g^Includes multiple-use patients

^h^Suvorexant and lemborexant.

## 4 Discussion

In this study, we used data on the PD rate and risk factors at a secondary hospital in Japan because there were some biases related to the clinical department and treatment policy. A retrospective cohort study was conducted to screen for PD risk factors. In this study, the incidence of PD after valve replacement or valvuloplasty was 25% (35/142 patients). Humbert et al. [11] reported that the incidence of PD in patients undergoing aortic valve replacement was 23%, which is consistent with this study. Moreover, the time-dependent frequency of PD exhibited general characteristics (Fig 3), suggesting that the assessment of PD in this study using ICDSC scoring was appropriate. Although age, benzodiazepine treatment, and duration of ICU stay were risk factors for PD in the univariate analyses, only polypharmacy was identified as an independent factor in the multivariate analysis (Table 2). Patients with polypharmacy tend to be elderly, have longer ICU stays, and may have used benzodiazepines at a higher frequency than those with non-polypharmacy, therefore, no significant differences were observed. Recently, several reports suggested that polypharmacy is an independent risk factor for PD in ventral hernia repair [25], hip fracture treatment [26], and spinal surgery [27], suggesting that polypharmacy may be associated with PD after elective valve replacement or valvuloplasty. A significant moderate correlation was observed between the number of medications and the number of comorbidities; therefore, it was suggested that polypharmacy reflects the multiple comorbidities. However, ROC analysis for PD prediction showed that the values of AUC for the number of medications was higher than that of the number of comorbidities. Additionally, the number of comorbidities was not an independent risk factor for PD (Table 2), suggesting that polypharmacy may be more strongly related to cardiovascular surgical PD than the number of comorbidities. Therefore, further studies are needed to clarify the relationship between PD and polypharmacy. Polypharmacy was adopted as a risk factor in this protocol, because it is an appropriate indicator that allows PD risk to be easily assessed by pharmacists. Additionally, a high number of doses to take was also correlated with polypharmacy, therefore, this factor was added.

The development of PD during cardiovascular surgery has been suggested to be associated with sleep disorder [28]. In this PBPM, the pharmacist recommends aggressive intervention with ramelteon and suvorexant for patients complaining of insomnia. It has been reported that the administration of suvorexant and ramelteon can also suppress PD [21]. In fact, the introduction of PBPM decreased the use of benzodiazepines and increased the use of ramelteon and orexin receptor antagonists (Table 3), suggesting that this PBPM may have decreased PD due to an increase in ramelteon and orexin receptor antagonists and not just benzodiazepine discontinuation. However, it is possible that the disproportion of surgical procedures during the pre- and post-PBPM periods may have affected the incidence of PD. Therefore, we focused on open-heart surgery as a subgroup analysis. Although a decreasing trend in PD incidence was observed in post-PBPM in patients who underwent open-heart surgery, a significant difference was not observed due to a limited sample size. Despite the introduction of PBPM, approximately 23% of the patients remained on benzodiazepine treatment (Table 3). In general, withdrawal symptoms are observed with multiple benzodiazepines and/or long-term administration for 6 months, and discontinuation of benzodiazepines is difficult [29]. Therefore, tapering is recommended for patients taking benzodiazepines for long periods or regularly [30]. Additionally, we believe that a reduction in the number of concomitant medications would be useful in reducing PD. However, since this protocol was introduced from one day before elective surgery, pharmacists could not actively intervene to reduce benzodiazepine use and polypharmacy. Taking these improvement points into consideration, pharmacists may be able to decrease benzodiazepine use and polypharmacy by intervening in the outpatient clinic when surgery is scheduled rather than the day before surgery. It has been well-known that not only drugs, which are direct factors, but also other promoting factors, such as pain and bedside environmental control, are involved in PD development [5]. It has been reported that medical team managing suppressed PD in cardiovascular surgery [31], therefore, we believe that this protocol can be further revised to include intervention points from other healthcare workers, including nurses, to further reduce the incidence of PD.

This study had several limitations. First, this was a single-center retrospective study, therefore, the efficacy of PBPM was influenced by biases related to the treatment policy of one center. Second, it was unclear whether healthcare workers were educated or trained in PD assessment, therefore, ICDSC scores may differ among evaluator. Additionally, ICDSC scores may have been underestimated after ICU discharge. Third, it would be appropriate to evaluate the effect of PBPM on the incidence of PD for each operative procedure; however, we could not elucidate this due to the small sample size. Forth, the degree of improvement in insomnia was not assessed using an objective measure, such as the Pittsburgh Sleep Quality Index. Therefore, the degree of improvement in sleep quality following PBPM remains unclear. Fifth, changes in personnel and the attitudes of healthcare workers toward PD and insomnia drug-prescribing trends over time may have led to an overestimation of the efficacy of the pharmacist-led intervention.

Overall, this study demonstrated that pharmacist-led interventions based on PBPM may contribute to reduce the PD in cardiovascular surgery.

## 5 Conclusion

This study shows that polypharmacy may be a strong predictor for PD in cardiovascular surgery. Moreover, the development of PD may be decreased by protocol-based pharmacist intervention, reducing benzodiazepine use, and including ramelteon and orexin receptor antagonists. Although the present PBPM still requires further modification, it is simple and potentially useful for pharmacists to assess the risk of patients undergoing any elective cardiovascular surgery. Few reports have demonstrated the clinical effectiveness of this approach, and we propose that these interventions provide a new strategy specific to PD attenuation.

## Supporting information

S1 Fig Correlation between the number of medications and the number of comorbidities. (PPTX)

S2 Fig ROC curve of the number of comorbidities for PD after valve replacement or valvuloplasty. (PPTX)

S1 Table Baseline clinical characteristics and laboratory data of eligible patients in pre-and post-PBPM via open-heart surgery. (DOCX)
