# Supplementary figures and images for: Effect of pharmacist-led intervention protocol on preventing postoperative delirium after elective cardiovascular surgery
Source: PLoS One. 2023 Oct 12;18(10):e0292786. doi: 10.1371/journal.pone.0292786 (PMC10569577; doi:10.1371/journal.pone.0292786)

## Slide 1
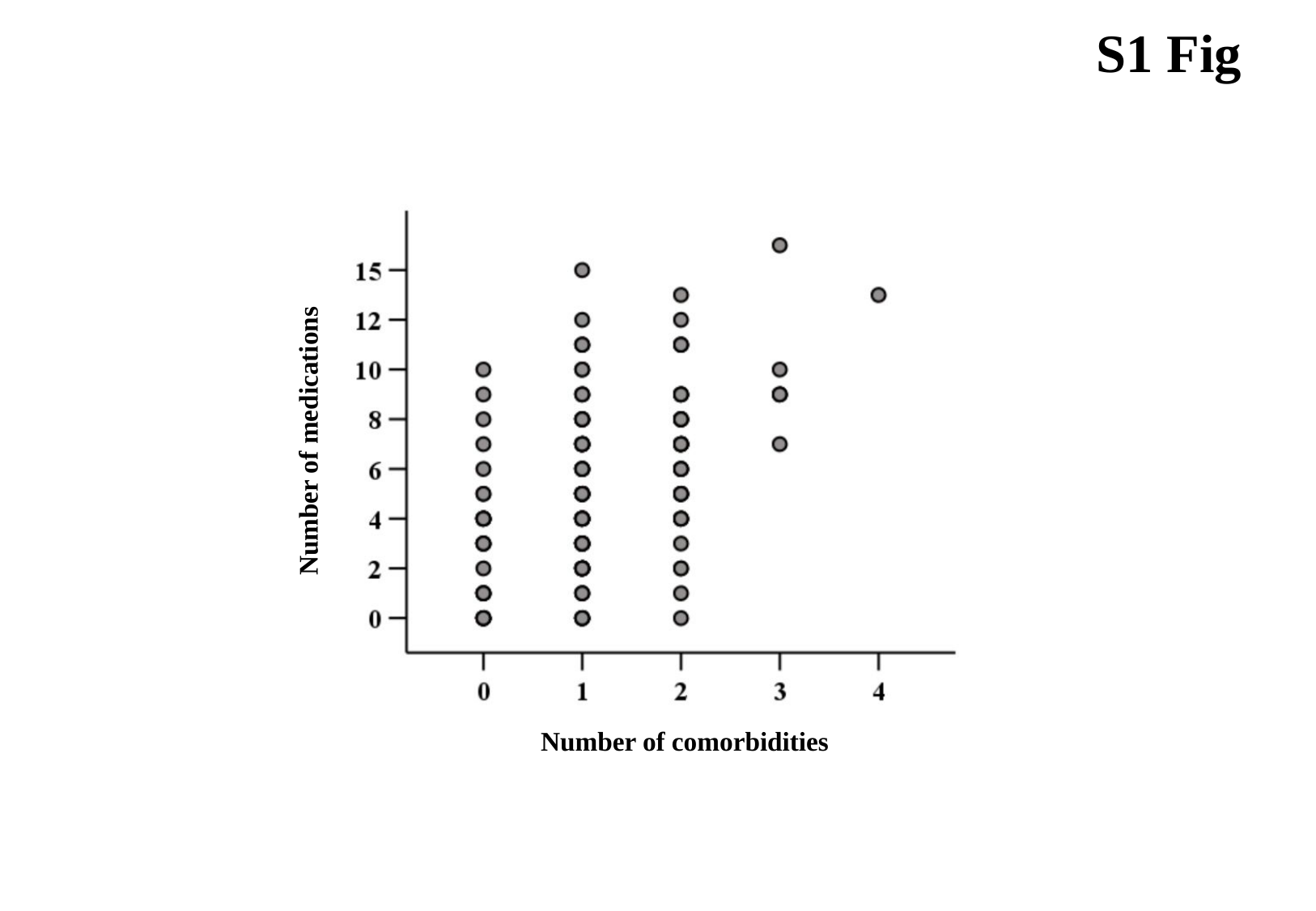

S1 Fig
Number of medications
Number of comorbidities

Supplement: S1 Fig — (PPTX) [file pone.0292786.s001.pptx]

## Slide 1
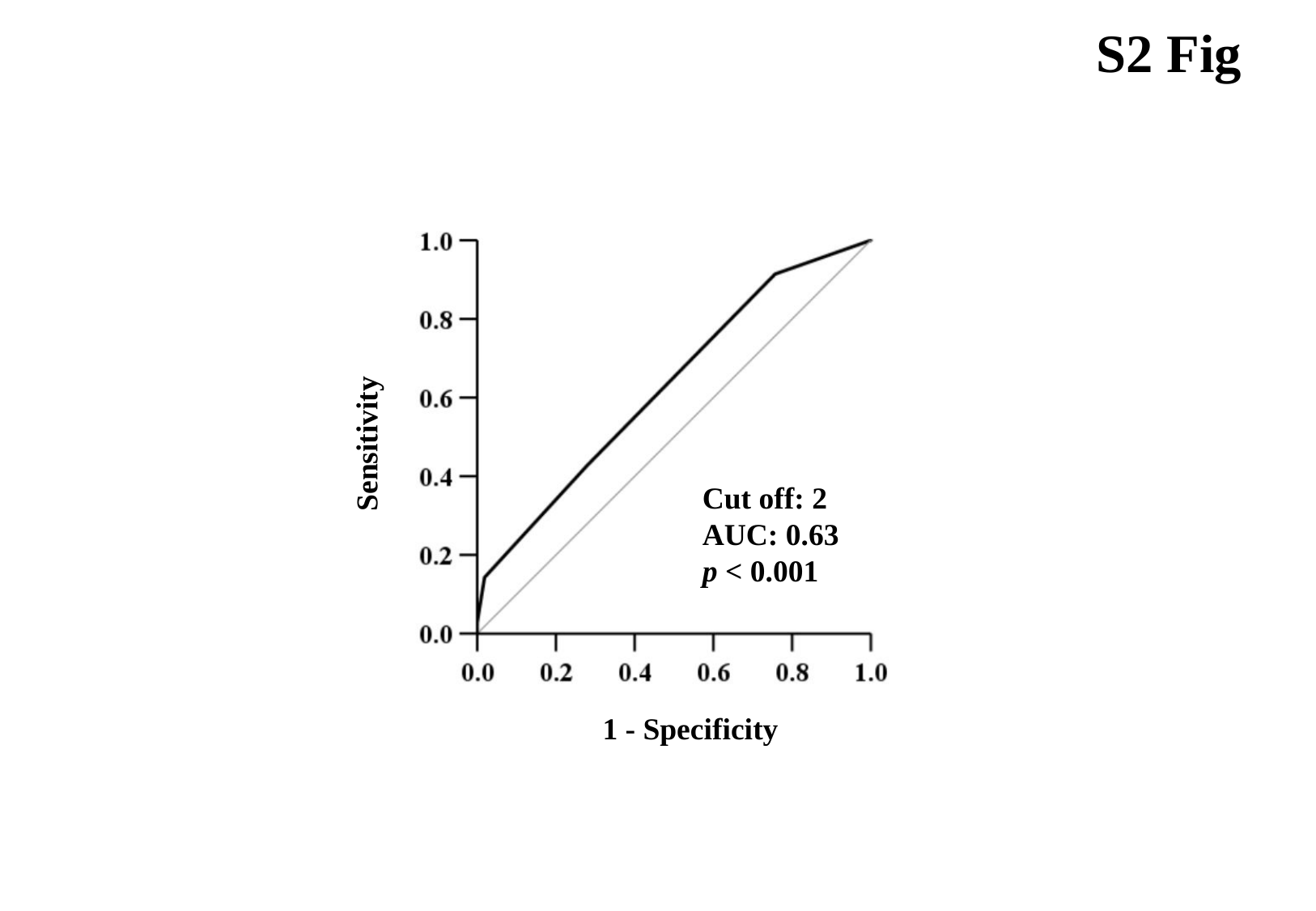

S2 Fig
Sensitivity
Cut off: 2
AUC: 0.63
p < 0.001
1 - Specificity

Supplement: S2 Fig — (PPTX) [file pone.0292786.s002.pptx]
