# Supplementary material for: Effect of pharmacist-led intervention protocol on preventing postoperative delirium after elective cardiovascular surgery
Source: PLoS One. 2023 Oct 12;18(10):e0292786. doi: 10.1371/journal.pone.0292786 (PMC10569577; doi:10.1371/journal.pone.0292786)
Supplement: S1 Table — (DOCX) [file pone.0292786.s003.docx]

**S1 Table. Baseline clinical characteristics and laboratory data of eligible patients in pre-and post-PBPM via open-heart surgery.**

| Factors | Pre-PBPM | Post-PBPM | *p* value |
| --- | --- | --- | --- |
|  | n = 34 | n = 39 |  |
| Preoperative Information |  |  |  |
| Basic property |  |  |  |
| Sex (Male/Female) | 22/12 | 31/8 | 0.16^a^ |
| Age (years) | 76 (70, 80)^f^ | 73 (68, 79)^f^ | 0.20^d^ |
| Body weight (kg) | 56 (49, 60)^f^ | 61 (54, 75)^f^ | 0.016^d^ |
| eGFR (mL/min) | 62 ± 21^e^ | 61 ± 20^e^ | 0.83^c^ |
| Albumin (g/dL) | 3.1 (2.5, 3.7)^f^ | 2.7 (2.4, 3.4)^f^ | 0.25^d^ |
| Total bilirubin (mg/dL) | 0.95 (0.78, 1.3)^f^ | 0.80 (0.55, 0.90)^f^ | 0.023^d^ |
| ALT (IU/L) | 16 (11, 24)^f^ | 16 (11, 27)^f^ | 0.83^d^ |
| Operation Procedure |  |  |  |
| CABG, n (%) | 12 (35) | 26 (67) | 0.007^a^ |
| Aortic root replacement, n (%) | 3 (9) | 4 (10) | 1.0^b^ |
| Valve replacement/Valvuloplasty, n (%) | 19 (56) | 9 (23) | 0.004^a^ |
| Oral medications |  |  |  |
| Number of medications | 9 (6, 11)^f^ | 9 (7, 11)^f^ | 0.67^d^ |
| Benzodiazepines, n (%) | 14 (41) | 13 (33) | 0.49^a^ |
| H_2_ blockers, n (%) | 2 (6) | 1 (3) | 0.60^b^ |
| Proton-pomp inhibitors, n (%) | 10 (29) | 24 (62) | 0.009^b^ |
| Steroids, n (%) | 3 (8.8) | 0 (0) | 0.096^b^ |
| *β* blockers, n (%) | 11 (32) | 14 (36) | 0.75^a^ |
| Anti-arrhythmic drugs, n (%) | 6 (18) | 0 (0) | 0.008^b^ |
| Anti-hypertensives, n (%) | 27 (79) | 34 (87) | 0.37^a^ |
| Dementia drugs, n (%) | 0 (0) | 1 (3) | 1.0^b^ |
| Anti-psychotics, n (%) | 0 (0) | 5 (13) | 0.057^b^ |
| Number of doses to take | 5 (4, 5)^f^ | 4 (4, 5)^f^ | 0.32^d^ |
| Postoperative Information |  |  |  |
| Operation time (min) | 328 (234, 414)^f^ | 295 (254, 349)^f^ | 0.64^d^ |
| Anesthesia time (min) | 388 (293, 480)^f^ | 353 (318, 431)^f^ | 0.56^d^ |
| Duration of ventilator management (day) | 7 (5, 7)^f^ | 6 (5, 7)^f^ | 0.68^d^ |
| Duration of ICU stay (day) | 4 (3, 5)^f^ | 4 (3, 5)^f^ | 0.83^d^ |
| SOFA score | 9 (6, 10)^f^ | 10 (8, 11)^f^ | 0.29^d^ |
| Dexmedetomidine treatment, n (%) | 20 (59) | 25 (64) | 0.64^a^ |
| Insomnia drugs^g^ use |  |  |  |
| Benzodiazepines, n (%) | 15 (44) | 9 (23) | 0.056^a^ |
| Ramelteon, n (%) | 10 (29) | 30 (77) | < 0.001^a^ |
| Orexin receptor antagonists^h^, n (%) | 5 (15) | 21 (54) | < 0.001^a^ |

ALT: alanine aminotransferase. CABG: coronary artery bypass grafting. eGFR: estimated glomerular filtration rate. ICU: intensive care unit. SOFA: sequential organ failure assessment.

^a^Chi-square test. ^b^Fisher's exact test. ^c^Student’s t-test. ^d^Mann-Whitney U test. ^e^Each value represents the mean±standard deviations. ^f^Each value represents the median (25th, 75th percentile). ^g^Includes multiple-use patients. ^h^Suvorexant and lemborexant.
